# Direction-dependent contributions of cardiac myofilament networks to myocardial passive stiffness reveal a major disparity for titin

**DOI:** 10.1007/s00395-025-01119-8

**Published:** 2025-06-13

**Authors:** Felix A. Wagner, Christine M. Loescher, Andreas Unger, Michel Kühn, Annika J. Klotz, Ivan Liashkovich, Dominika Ciechanska, Hermann Schillers, Franziska Koser, Johanna K. Freundt, Anthony L. Hessel, Wolfgang A. Linke

**Affiliations:** Institute of Physiology II, University of Münster, Robert-Koch-Str. 27B, 48149 Münster, Germany

**Keywords:** Myocardium, Titin, Actin, Myosin, Passive stiffness, Atomic force microscopy

## Abstract

Progressive myocardial dysfunction in patients with heart failure often involves alterations in myocardial passive stiffness, yet the underlying mechanisms remain incompletely understood. While passive stiffness in the longitudinal direction has been extensively characterized via uniaxial tensile stretching of cardiac specimens, transverse stiffness has received far less attention despite its equal mechanical importance. In this study, we combined atomic force microscopy nanoindentation with stretching assays on myocardial preparations to quantify the relative contributions of the three myofilament networks – actin, myosin, and titin – to passive stiffness in both transverse and longitudinal orientations. We employed a transgenic mouse model in which titin’s elastic springs contain a tobacco etch virus protease (TEVp) recognition site, enabling selective and acute titin cleavage upon TEVp treatment. Actin filaments were severed using a calcium-independent gelsolin fragment, and myosin filaments were dissociated by high-salt extraction. Along the longitudinal axis, titin accounted for over 50% of total passive stiffness in both cardiac fiber bundles and isolated cardiomyocytes across most physiological strain ranges, whereas actin contributed under 35% overall – and only 15–20% within the collagen-containing fiber bundles. In contrast, in the transverse axis, titin and actin each contributed approximately 20–26% of passive stiffness in cardiac slices under varying compression forces. The myosin-titin composite thick-filament network contributed ~ 55% longitudinally but only ~ 35% transversely. These results reveal pronounced, direction-dependent differences in myofilament contributions to myocardial passive stiffness, with titin exhibiting the greatest disparity. Our findings deepen our understanding of the myocardium’s multidimensional mechanics and may inform therapeutic strategies to ameliorate pathological cardiac stiffening.

**Supplementary Information:**

The online version contains supplementary material available at 10.1007/s00395-025-01119-8.

## Introduction

Functional remodeling in heart failure often involves altered myocardial passive stiffness, as observed in heart failure with preserved ejection fraction (HFpEF) [26, 30, 46]. HFpEF hearts exhibit increased passive stiffness compared to healthy controls, evident at multiple scales, from whole-heart measurements to isolated cardiac preparations [15, 62, 63, 67, 68]. This stiffening arises from changes in the extracellular matrix (ECM), particularly collagen, and in cardiomyocyte proteins [16, 51, 65, 68]. Within cardiomyocytes, sarcomeres play a critical role in determining passive stiffness. These contractile units comprise three main myofilament systems – myosin-based thick filaments, actin-based thin filaments, and elastic titin filaments – responsible for both active and passive forces [53, 56]. Titin is a key determinant of myocardial passive stiffness [31, 34, 39, 40] and also modulates active force via mechanisms such as length-dependent activation (the Frank-Starling law) [22, 34]. Alterations in titin isoform expression and posttranslational modifications, including phosphorylation, oxidation, and acetylation, contribute to myocardial stiffening [40].

Multidirectional forces acting on myocardial fibers within the ventricular wall influence both active force development and relaxation, and also affect diastolic passive stiffness. Most *ex vivo* studies investigating contributors to myocardial passive stiffness have focused on longitudinal stiffness using uniaxial tensile‐stretch experiments. These studies show that titin is the primary determinant of longitudinal stiffness, with additional contributions from cytoskeletal filaments and the ECM [18, 38, 39]. However, transverse stiffness is equally relevant yet far less studied [9, 25, 49]. Quantifying the contributions of individual myocardial structural elements to both longitudinal and transverse passive stiffness is essential for understanding tension development and transmission during diastole and systole. Notably, although “stiffness” is commonly used to describe the mechanical properties of tissues and other solids, the term is often neither clearly defined nor appropriately applied [14]. Here, we define “stiffness” in terms of passive force-extension or passive force-compression relationships (i.e., stress–strain or stress–indentation curves), using it synonymously with “elasticity,” quantified as the elastic (Young’s) modulus in Pascals.

In this study, we investigated the individual contributions of the three principal myofilament networks to transverse passive stiffness and compared them with their roles in the longitudinal direction. Myosin filaments were disrupted using high-salt extraction (1 M KCl), whereas actin filaments were severed with the Ca^2+^-independent gelsolin fragment GLN-40 [17, 21, 37]. To overcome the limitations of previous titin-targeting methods [35], we employed the titin cleavage (TC) mouse model [48], which harbors a tobacco etch virus protease (TEVp) recognition site in titin’s elastic region and a HaloTag for labeling. Introduction of TEVp enabled targeted, complete (100%) cleavage of titin springs in homozygous (Hom) TC mouse heart tissue [39]. Using this model, we performed atomic force microscopy (AFM) nanoindentation on left ventricular (LV) tissue sections to quantify transverse passive stiffness, and tensile stretching of cardiac specimens to measure longitudinal stiffness. Our results show that myosin-titin composite filaments make major contributions to passive stiffness in both transverse and longitudinal directions, whereas titin alone contributes less than half as much transversely as it does longitudinally. In contrast, actin’s contribution is slightly greater in the transverse axis than in the longitudinal. These findings refine our understanding of cardiac stiffness, inform *in silico* whole-heart simulations, and offer insights for translational studies aimed at modulating heart stiffness in vivo, particularly in HFpEF.

## Methods

### Animals

We performed all experiments on cardiac tissue samples from *Mus musculus* with the background strain C57BL/6JRj. We obtained permission for all procedures from the local animal welfare authority (Landesamt für Natur, Umwelt und Verbraucherschutz Nordrhein-Westfalen, LANUV NRW, 81-02.04.2019.A472). A total of N = 28 Hom TC mice and N = 21 wild-type (WT) mice were used for the experiments. Mice were bred at Münster University Hospital and reared with ad libitum access to food and water, following a 12-h day-night cycle. At all times, we adhered to the local guidelines for animal handling and performed all animal studies in accordance with the ethical standards laid down in the 1964 Declaration of Helsinki and its later amendments. To obtain a mouse heart for mechanical or biochemical experiments, we sacrificed the mice by cervical dislocation. The heart was immediately removed, and we proceeded with the experiment-specific preparations.

### In-house production of TEVp

For the production of TEVp, we followed our published protocol [32, 39]. Briefly, using vector pMHT 238 Delta, we performed heat shock-mediated transformation of BLR (DE3) cells. When the OD600 reached 0.6 to 1.0, we incubated the cells for 3 h with 1 mM isopropyl-β-D-thiogalactopyranoside at 37 °C to activate the bacterial protein synthesis machinery and initiate extensive protein expression. We captured the expressed TEVp proteins with Ni-NTA (Qiagen, 30210) and, after elution with 250 mM imidazole, stored the TEVp in relaxing solution (170 mM potassium propionate, 20 mM 3-(N-morpholino) propane sulfonic acid, 2.5 mM magnesium acetate, 5 mM K_2_EGTA, 2.5 mM ATP, 14.5 mM creatine phosphate) with 1 × working concentration protease inhibitor (1 × PI, Promega G6521). We refer to this relaxing solution as iRS. For the stretch experiments on glycerinated fiber bundles, we used commercial TEVp (ThermoFisher Scientific, 12575–023) instead of the in-house produced TEVp.

### Preparation of cardiac sections for AFM measurements

In preliminary experiments, we conducted AFM nanoindentation measurements on permeabilized single isolated LV cardiomyocytes. Despite coating the cover glass with glue, these cells slightly moved during indentation, resulting in unstable force signals. Consequently, we decided to use only cardiac sections for the transverse stiffness measurements. To this end, we manually removed the heart from a sacrificed mouse and perfused the left ventricle retrogradely with phosphate-buffered saline (PBS) for 15–20 min. The entire heart was then transferred into PBS enriched with 1 × PI (“working PBS”). We used only one-half of the heart for sectioning, ensuring that the LV tissue contained trabeculae. The tissue was embedded in 2% agarose and the horizontal alignment of the trabecular fibers was verified under a microscope (Stemi 2000, Zeiss). With a vibratome (VT1000 S, Leica), we cut 20 µm thin sections from the agarose block, selecting only those with trabecular muscle for further analysis. Throughout the sectioning process, we ensured that the cardiac muscle fibers were aligned parallel to the vibratome blade and were continuously covered with PBS. Section quality and fiber alignment were again controlled with a microscope. The sections were stored in working PBS at 4 °C for up to 4 days.

Before AFM measurements, each section was incubated for 20 min in working rigor solution (RiS) supplemented with 0.5% Triton-X-100. The working RiS consisted of RiS (100 mM KCl, 50 mM Tris, 2 mM MgCl_2_, 1 mM EGTA, pH 7.0), 10 mM 2,3-butanedione monoxime (BDM) and 1 × PI. After incubation, each sample was washed several times: twice with working RiS and twice with working RS (wRS), which included RS with 10 mM BDM and 1 × PI. The section was then attached to the center of a coverslip that had previously been coated with Cell-Tak (2.52 mg/ml, Corning, 354240) and washed with ethanol, water, and wRS, ensuring that the section was completely covered with wRS, before gently pressing it down with a second coverslip for 15 min. All procedures were performed at 4 °C.

### Transverse stiffness measurements by AFM nanoindentation

Nanoindentation experiments were performed using an atomic force microscope (Nanowizard 3, JPK Instruments, Berlin) in contact mode. Measurements were carried out at room temperature in closed-loop mode with drift-compensated probes (MLCT-SPH-5UM-DC, 4.9 µm tip radius; Bruker). Tip velocity was set to 1 µm/s, and the maximum loading force applied was 10 nN. Cantilever spring constants ranged from 0.224 to 0.407 N/m, as determined by an interferometer (OFV-551; Polytec, Waldbronn, Germany). Deflection sensitivity was calibrated following the Standardized Nanomechanical Atomic Force Microscopy Procedure [7, 54].

After completing a control set of measurements in wRS, we treated the samples with different chemicals depending on the experimental question: (1) 60-min incubation with wRS containing 50 µg GLN-40 (8305-01, Hypermol) for actin severing; (2) 25-min treatment with wRS and TEVp to achieve complete titin cleavage (Hom TC mice), with WT mice samples as controls; (3) 10-min treatment with wRS and 1 M KCl for thick filament extraction experiments; and (4) 10-min or 60-min treatment with pure wRS for control measurements in experiments (1) and (3). At the end of each incubation, a washing step was performed, before conducting a second set of measurements in wRS at the original locations. We also used a subset of GLN-40-treated samples and incubated them with wRS and 1 M KCl for 10 min after completing the second measurement round to study the combined effects of actin severing and thick filament extraction.

Additionally, we performed AFM measurements in force mapping mode to study the impact of biological and technical variability on our quantifications. To this end, we repeatedly conducted stiffness measurements on grids of 6 × 6 pixels with 5 µm inter-pixel spacing. Each grid was measured three times, using the same settings as described above.

To extract individual stiffness values from the AFM measurements, we analyzed each recording using Punias (Protein Unfolding and Nano-Indentation Analysis Software, version 1.0, release 2.3, http://punias.free.fr). Using this software, we applied the linearized form of the Hertz model to each force curve [7] at two distinct compression forces, 2.00–2.25 nN and 4.00–4.25 nN, according to:

1 $$E = \frac{3}{4}*\left( {\frac{{\Delta F^{\frac{2}{3}} }}{\Delta d}} \right)^{\frac{3}{2}} *\frac{{1 - \nu^{2} }}{\sqrt r }$$

This equation enabled us to calculate the Young’s modulus (*E*) by analyzing the force range (Δ*F*, 0.25 nN in our experiments) and the corresponding indentation range (Δ*d*) in a force-distance curve. The other parameters are the Poisson ratio (*ν*), for which we used a value of 0.5, and the spherical tip radius (*r*). In this way, we calculated the Young’s modulus as a measure of transverse stiffness.

Results were visualized in two ways: (i) to demonstrate how a particular treatment altered stiffness, we plotted post-treatment *E*-values relative to corresponding values before treatment; and (ii) to illustrate variability between measurements, we showed *E*-values after treatment relative to the pre-treatment median. Relative changes in stiffness were expressed as a percentage (%).

Exploratory, unpaired measurements indicated that incubation with iRS plus Triton X-100, used to permeabilize membranes, did not significantly change the average transverse stiffness of TC mouse cardiac tissue sections (Fig. S1).

### Longitudinal force measurements on cardiac fibers

We performed force-extension measurements in the (non-activated) state on three types of multicellular cardiac preparations: (1) freshly prepared cardiac fiber bundles; (2) fiber bundles stored at  – 20 °C in glycerol-containing buffer (glycerinated fibers); and (3) frozen fiber bundles, all of which were membrane-permeabilized. For details, see [39].

*Freshly prepared fiber bundles:* LV fibers encompassing papillary muscles or trabeculae were excised from mouse hearts, cleared of blood, and extensively washed with normal Tyrode’s solution (140 mM NaCl, 0.5 mM MgCl_2_, 0.33 mM NaH_2_PO_4_, 5 mM HEPES, 5.5 mM glucose, 5 mM KCl, pH 7.4). Mechanical experiments were conducted at 37 °C, with fibers continuously covered in solution. Fibers were permeabilized using modified iRS (170 mM sodium propionate and 0.5% Triton-X-100) for at least 30 min, followed by two washes with iRS. Using a setup combining a micromotor and a force transducer (Scientific Instruments, Heidelberg, Germany), we stretched the fiber bundles (1.0–2.5 mm length; 0.1–0.4 mm thickness; attached to aluminum clamps) in five steps from slack length to 20% strain. After each stretch, we observed a 10-s hold period before returning the fiber to slack, whereas in between stretch-release cycles, we waited 1 min. For any step amplitude, the cycle was performed twice. Force recordings were made at 1,000 Hz using customized software (https://github.com/DrDJIng/FiberStretchProgram). Only peak forces at each strain level were analyzed. After control measurements, fibers were incubated with 50 µg GLN-40 in iRS for 30 min to sever actin filaments, and passive force measurements were repeated. Strain-dependent peak forces were normalized to the maximum peak force at the longest stretch during control conditions, and data were fitted with a second-order polynomial.

*Glycerinated cardiac fiber bundles:* Excised TC mouse hearts were retrogradely perfused with a rigor solution (75 mM KCl, 2 mM MgCl_2_, 2 mM EGTA, 10 mM Tris, pH 7.1) containing 50% glycerol for approximately 15 min. They were then stored at  – 20 °C with added protease inhibitor (one tablet per 100 ml, Roche Diagnostics) for a minimum of 4 weeks before use in measurements. Pre-permeabilized cardiac fiber bundles were attached to the force measurement setup (Scientific Instruments, Heidelberg, Germany). After extensive washing with iRS, force recordings were made at room temperature while stretching the fibers in six steps from starting length (~ 1.8 µm sarcomere length (SL)) to 30% strain. Each step was held for 10 s before proceeding to the next level. After the last stretch, fibers were returned to starting length and rested for at least 5 min before repeating the stretching cycle until differences between cycles became negligible (usually after 2–3 rounds). Data from the last cycle were used for analyses. Fibers were incubated in TEVp for 10 min, and passive forces were recorded again. Final analyses utilized peak forces from each step before and after TEVp incubation, normalized to the average peak force at 25% strain in control measurements, and fitted with a second-order polynomial function.

*High-salt experiments on thawed fiber bundles:* WT fiber bundles from frozen mouse hearts were dissected, washed with wRS, and permeabilized with 1.5% Triton-X-100 at 4 °C overnight. After washing, stretching experiments were conducted similarly to those on glycerinated fibers, but with 60 s of stress relaxation at each level and wRS instead of iRS. After control measurements, fibers were incubated with 1 M KCl for 60 min, washed, and then post-treatment stretching was performed.

Treatment-dependent relative changes in force at a given strain as a measure of stiffness decrease were expressed as a percentage (%).

### Tensile force measurements on single cardiomyocytes

For detailed protocols on tensile stretching of single cardiomyocytes, see [39]. Briefly, cardiomyocytes were harvested from LV tissue pieces of Hom TC mouse hearts previously frozen at  – 80 °C. After mechanical homogenization in iRS, the cells were incubated for 8 min in iRS and 0.5% Triton-X-100 for permeabilization, followed by three washes with iRS to remove Triton. Mechanical experiments used a setup (Aurora Scientific, 403A) that combines a sensitive force transducer and a piezo motor, with a cardiomyocyte suspended using Shellac glue (in 70% ethanol). During measurements, cells were observed under an Axiovert 135 inverted microscope (Carl Zeiss) for SL recording.

The measurement protocol involved stretching the cardiomyocytes from slack (~ 1.85 µm SL) by ~ 20%, holding for 7 s, and then returning to slack while continuously measuring forces. The cardiomyocytes were fully covered with iRS throughout the procedure. Each measurement was repeated three times, and the mean peak force of these repetitions was analyzed. Following control measurements, we performed one of the following treatments: (1) a 10-min incubation with iRS containing TEVp, (2) a 30-min incubation with iRS containing 25 µg GLN-40, or (3) a 10-min incubation of WT cardiomyocytes with iRS and TEVp as an additional control. Force changes were normalized to the corresponding average peak force before incubation and median changes were expressed in %.

### Confocal microscopy

Tissue integrity and the success of protein extraction were assessed by immunofluorescence (IF) microscopy. For one approach, we preserved TC mouse cardiac sections after AFM mechanical measurements using PBS enriched with 4% paraformaldehyde and 15% picric acid (pH 7.4), storing samples at 4 °C for several months. For IF microscopy, sections were washed twice with PBS and incubated for one hour in PBS with 5% bovine serum albumin (BSA) and 0.5% Trition-X-100. They were then transferred to a PBS solution with 0.5% BSA containing primary antibodies: anti-α-actinin (EA-53, Sigma-Aldrich) at 1:100 and anti-α-myosin heavy chain (22281-1-AP, Proteintech) at 1:250, and incubated overnight at 4 °C. The next day, unbound proteins were removed by three times washing with PBS, and samples were incubated overnight at 4 °C with secondary antibodies: Cy3-conjugated IgG (Jackson ImmunoResearch) at 1:400 and AlexaFluor-488 conjugated IgG (Invitrogen) at 1:400. Phalloidin-Alexa-647 (A22287, Invitrogen) at 1:50 was included for actin labeling. After washing out unbound proteins, we used a Nikon ECLIPSE Ti2 (Nikon Europe, Amstelveen) confocal microscope to assess protein structure and integrity, processing images with Image J (version 1.54f, NIH). The same treatment protocol and analysis approach (including microscopy settings) were applied throughout the procedures to ensure reproducibility.

In an alternative approach, we performed IF microscopy and AFM nanoindentation simultaneously. The atomic force microscope head was placed on the stage of a matching confocal microscope (TCS SP8, Leica, Wetzlar). We incubated sections for 15 min at 37 °C with a combination of iRS and 1:1000 diluted AlexaFluor-488-conjugated HaloLigand (Promega), then washed out unbound HaloLigand with iRS. HaloLigand binds covalently to the HaloTag within the genetic cassette of the TC mouse tissue, allowing visualization of the effect of titin cleavage within the cardiac tissue.

### Protein gel electrophoresis

We performed polyacrylamide gel electrophoresis (PAGE) on both GLN-40 and TEVp-treated samples, following published protocols [39, 45]. For GLN-40 treated samples, small tissue pieces were permeabilized by incubating them in iRS with 0.5% Triton-X-100 for 12 min. After washing out Triton with pure iRS, the sections were incubated in iRS with 25 µg GLN-40 at 4 °C for two hours. Corresponding controls were incubated under the same conditions without GLN-40. The pellet fraction was separated from the supernatant to qualitatively assess actin severing. The pellet was washed repeatedly with iRS and dissolved in modified Laemmli buffer (8 M urea, 2 M thiourea, 3% sodium dodecyl sulfate (SDS), 0.03% Serva Blue, 50 mM Tris HCl, pH 6.8, 10% glycerol, 75 mM dithiothreitol (DTT)). The supernatant was diluted 1:1 with modified Laemmli buffer. Both supernatant and pellet were incubated for 30 min at 0 °C, followed by 3 min at 97 °C. The samples were centrifuged at 18,407 × g for 3 min, and both fractions were separated by 10% PAGE.

For titin gels, small cardiac tissue pieces from frozen Hom and WT mouse hearts were incubated in iRS with 0.5% Triton-X-100 for 7 min. Triton was removed by washing three times with iRS. Half of the Hom and WT tissue was incubated for several hours with iRS containing TEVp and 100 mM DTT at 22 °C, while the other half was incubated similarly but without TEVp. All samples were dissolved in modified Laemmli buffer and incubated on ice for 20 min. They were then treated as described for the actin gels and separated by 1.8% PAGE. Protein bands on all PAGE gels were stained with Coomassie blue.

### Statistics

We used SPSS (version 29.0.2.0 (20)) and GraphPad Prism (version 9.5.1/10.4.1) for statistical testing and data transformation, GraphPad Prism for drawing diagrams. We set the significance level to 0.05. We checked for normality using the Shapiro–Wilk test and quantile–quantile plot visualization, and applied the best Box-Cox transformation to the response variables when required. We verified homoscedasticity and linearity of the (transformed) responses and performed residual diagnostics as necessary. To account for multiple comparisons, we applied a Bonferroni adjustment.

To assess contributions to transverse stiffness, we first screened for outliers using Tukey’s rule and the ROUT method (Q = 1%) [41]. Detected values were then examined, and those lacking a linear or logarithmic relationship with the remainder of the dataset were excluded. This approach accounts for erroneous measurements arising from unmatched measurement locations before and after treatment – an unavoidable issue given construction constraints. Importantly, repeating all statistical analyses on the full dataset (including outliers) produced comparable significance levels.

For statistical analyses, we fitted linear mixed-effects models (LME) using ANOVA frameworks. In the AFM-based transverse stiffness assays, “treatment” (before vs. after) and “compression force” (2 nN vs. 4 nN) were specified as fixed effects, while “location” (variability among measurement sites) was included as a random effect. Here, n denotes the number of measurement events (distinct locations × compression levels) and N the number of tissue slices.

Exploratory, unpaired comparisons of transverse stiffness before and after Triton X-100 treatment were performed using the Mann–Whitney test.

For stretch-induced peak force measurements in cardiac fiber bundles, we again employed an LME model, with “treatment” (before vs. after) and “strain” (4%, 8%, 12%, 16%, 20% or 5%, 10%, 15%, 20%, 25%) as fixed effects and “fiber variability” as a random effect. In this context, n corresponds to the total number of measurements and N to the number of animals.

In isolated cardiomyocyte stretch experiments, relative treatment effects (post/pre × 100%) were tested against the 100% baseline using a one-sample t-test. Here, n is the number of cardiomyocytes analyzed and N the number of mice.

All data are reported as median ± 95% confidence interval (CI).

## Results

### Variability of transverse passive stiffness across cardiac tissue sections

**Fig. 1:**
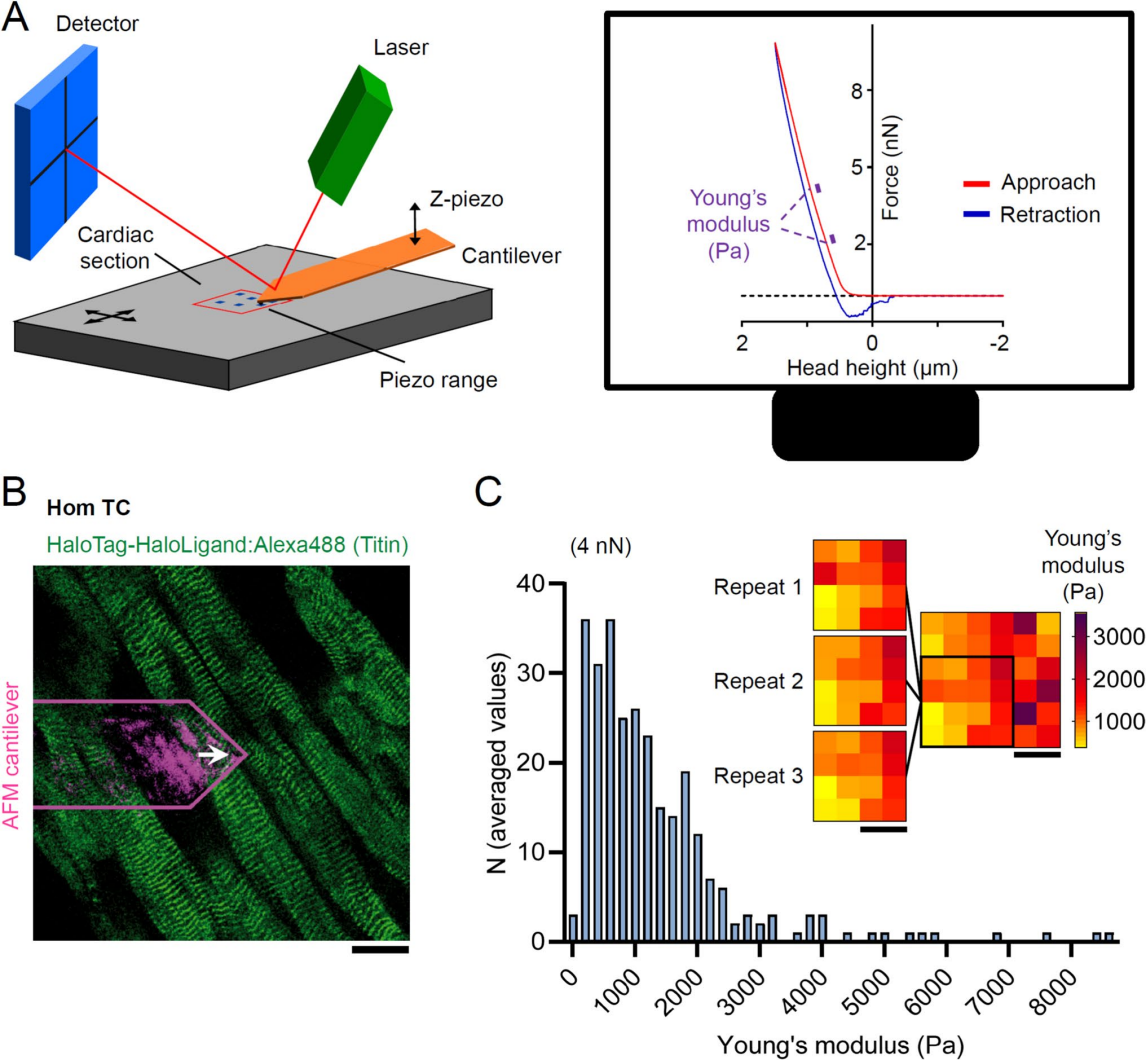
Measuring transverse passive stiffness in myocardial tissue slices with atomic force microscopy (AFM).** A** Schematic of the AFM setup (left), with blue dots marking measurement points in the red-lined piezo range, and representative force-height curve (right), with Young’s modulus calculated from the 2.00–2.25 nN and 4.00–4.25 nN force ranges (purple). **B** Representative fluorescence image of cardiac tissue section from a homozygous (Hom) titin-cleavage (TC) mouse labeled for titin with HaloLigand:AlexaFluor-488; the AFM cantilever (here, for demonstration purposes, Arrow-TL1, Nanoworld) and indenter position (white arrow) are indicated. Scale bar, 20 µm. **C** Histogram summarizing local Young’s moduli recorded in wRS (*n* = 280 locations, *N* = 33 slices) at 4.00–4.25 nN, with an inset heat map of repeated measurements (Repeats 1–3) of the same region and mean values; scale bars, 10 µm

We first sought to establish a robust AFM nanoindentation protocol for quantifying transverse passive stiffness in permeabilized TC mouse cardiac slices. Our primary measure was the Young’s modulus at indentation forces of 2.00–2.25 nN and 4.00–4.25 nN (Fig. 1A). Slices were prepared from LV trabecular muscle, whose fibers run predominantly in parallel (Fig. 1B), and stiffness profiles were acquired at multiple sites by averaging ~ 7 indentations per location. Under baseline (pre-disruption) conditions, transverse passive stiffness exhibited a non-normal distribution, with a median of 963.3 Pa and an interquartile range of 1215.2 Pa at 4.00–4.25 nN (Fig. 1C). This considerable variability persisted within and between sections. Importantly, AFM force-mapping controls (Fig. 1C, inset) ruled out technical fluctuations as the primary source of heterogeneity; instead, stiffness differences between measurement points appear to reflect intrinsic myocardial tissue heterogeneity.

### Thin filament contribution to transverse versus longitudinal passive stiffness

**Fig. 2:**
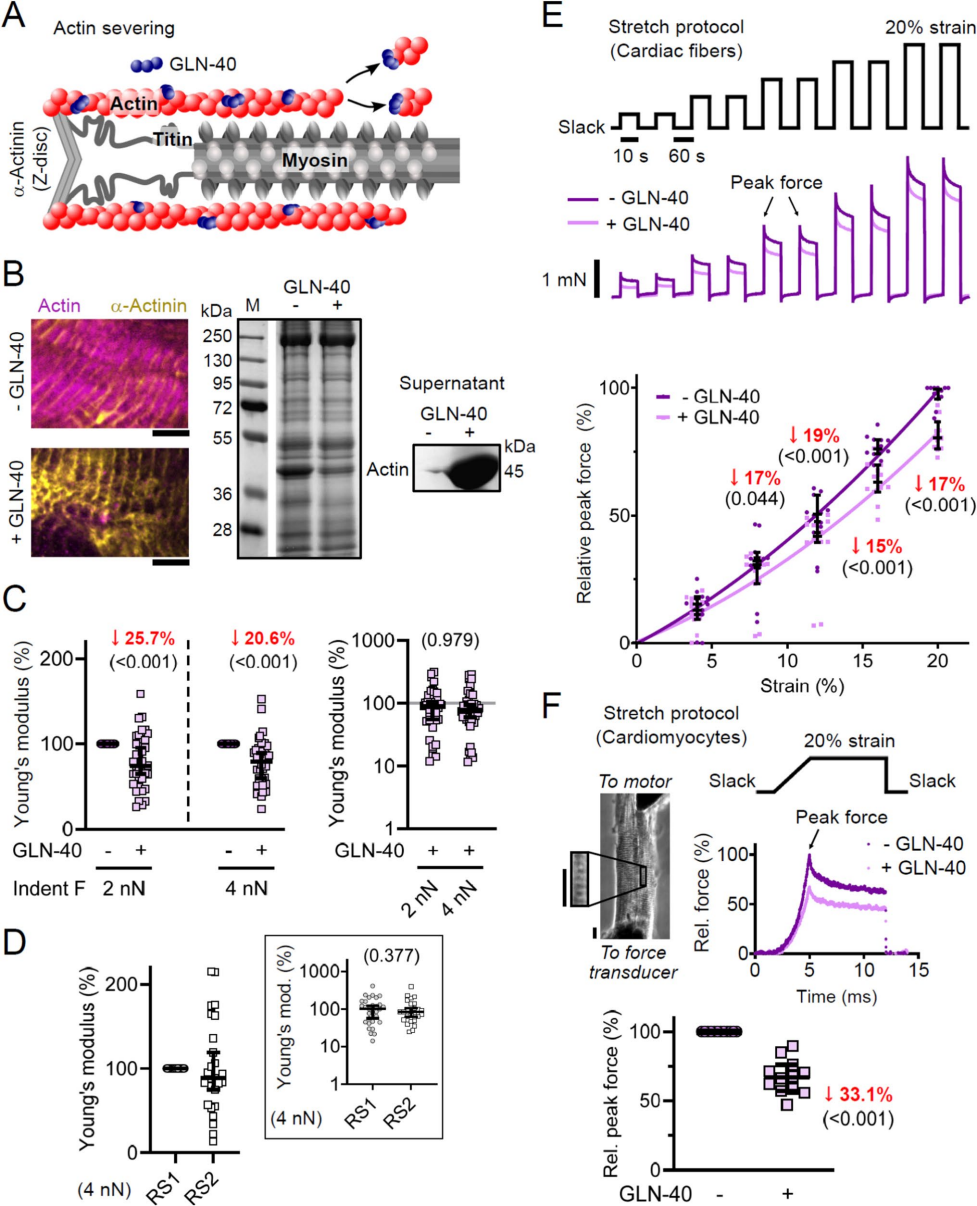
Contribution of actin filaments to transverse and longitudinal passive stiffness. **A** Schematic of GLN-40-mediated actin severing. **B** Permeabilized mouse LV cardiac samples treated for 60 min in wRS ± 50 µg GLN-40 and imaged by IF microscopy (left) using antibodies to actin (phalloidin-Alexa-647, magenta) and α-actinin (EA53 + AlexaFluor-488, yellow); scale bars, 5 µm. SDS-PAGE (right): homogenized TC fibers incubated for 2 h in iRS ± 25 µg GLN-40, pellet and supernatant separated, Coomassie-stained. **C** AFM nanoindentation of permeabilized WT cardiac slices (*n* = 78 measurements, *N* = 4 slices) before/after 60 min wRS + 50 µg GLN-40. Left: post/pre Young’s modulus at 2.00–2.25 nN and 4.00–4.25 nN indentation force; right: Young’s modulus (logarithmic scale) for GLN-40-treated samples, normalized to median pre-treatment. Data are median ± 95% CI. LME ANOVA with treatment and force level as fixed factors, measurement location as random factor. **D** AFM of WT slices (*n* = 30 measurements, *N* = 3 slices) before/after 60 min wRS alone (RS1 vs. RS2; these represent the same wRS but at different time points), recorded at 4.00–4.25 nN indentation force. Inset: Young’s modulus (logarithmic scale) relative to median RS1 condition. Data are median ± 95% CI. LME ANOVA with treatment as fixed factor, location random. **E** Passive force-extension on permeabilized TC cardiac fiber bundles (*n* = 12 measurements, N = 6 mice) before/after 30 min iRS + 50 µg GLN-40. Top: stretch protocol; middle: representative traces; bottom: peak force (median ± 95% CI) normalized to pre-treatment maximum, with second-order polynomial fits. LME ANOVA with treatment and strain (4–20%) as fixed factors, fiber as random factor; significant median changes in red (*p* in parentheses). **F** Passive force of permeabilized isolated cardiomyocytes before/after 30 min iRS + 25 µg GLN-40. Top: stretch protocol; middle left: phase image with ROI used for sarcomere length measurements (scale bars, 20 µm); middle right: force traces; bottom: average peak force (median ± 95% CI; *n* = 12 cardiomyocytes, *N* = 5 mice) normalized to pre-treatment. One-sample t-test; significant median change in red (*p *in parenthesis)

To assess the contribution of thin filaments to transverse passive stiffness, we applied our established AFM nanoindentation protocol to permeabilized mouse cardiac slices. Measurements were conducted before and after targeted actin severing, achieved by incubating samples in wRS supplemented with 50 µg of the gelsolin fragment GLN-40 (Fig. 2A), which disrupts both intra- and extra-sarcomeric actin filaments [17]. Successful actin depletion was confirmed by IF microscopy and protein gel electrophoresis (Fig. 2B). IF microscopy was performed on the same slices used for AFM, revealing near-complete removal of the actin network in GLN-40-treated samples compared to untreated controls (Fig. 2B, left), with only minor residual actin localized at and between Z-discs, in agreement with previous reports [37, 39]. Protein gel electrophoresis of larger tissue specimens demonstrated reduced actin in the centrifuged cell pellet and increased actin in the supernatant following GLN-40 treatment (Fig. 2B, right). AFM nanoindentation revealed a significant decrease in transverse passive stiffness after 60 min of GLN-40 incubation: the Young’s modulus fell by a median of 25.7% (95% CI 4.6–35.3%) at 2.00–2.25 nN indentation force and by 20.6% (95% CI 10.5–40.1%) at 4.00–4.25 nN (Fig. 2C; Fig. S2A). In contrast, control slices repeatedly incubated with wRS alone showed no significant change in transverse passive stiffness (Fig. 2D).

To compare thin-filament contributions to transverse versus longitudinal passive stiffness, we performed tensile stretching on permeabilized cardiac fiber bundles from Hom TC mouse left ventricle in relaxing buffer (Fig. 2E). Peak forces were recorded at incremental stretches from near-slack to 20% strain and expressed as a percentage of each fiber’s maximum force in the untreated control. A 30-min GLN-40 incubation (sufficient to deplete the fibers of actin [39]) reduced longitudinal passive force by 16.5% (1.7–28.7%), 18.7% (13.5–22.8%), 15.2% (11.7–28.7%), and 17.3% (12.3–21.0%) at 8%, 12%, 16%, and 20% strain, respectively (Fig. 2E, bottom). Together, these data indicate that actin-based thin filaments contribute slightly more to transverse than to longitudinal passive stiffness.

Since cardiac fiber bundles retain ECM components (e.g., collagen) that contribute to passive stiffness, we additionally isolated the actin-based component by measuring passive stretch force in single permeabilized TC cardiomyocytes (Fig. 2F). Using a one-step stretch from slack to ~ 20% cell strain (approximately 1.85–2.20 µm SL), a 30-min treatment with 25 µg GLN-40 reduced peak passive force by 33.1% (24.0–42.9%) (Fig. 2F, bottom). The larger relative decrease in isolated cardiomyocytes compared to fiber bundles reflects the absence of ECM stiffness, consistent with previous findings [39].

### Elastic titin contribution to transverse versus longitudinal passive stiffness

**Fig. 3:**
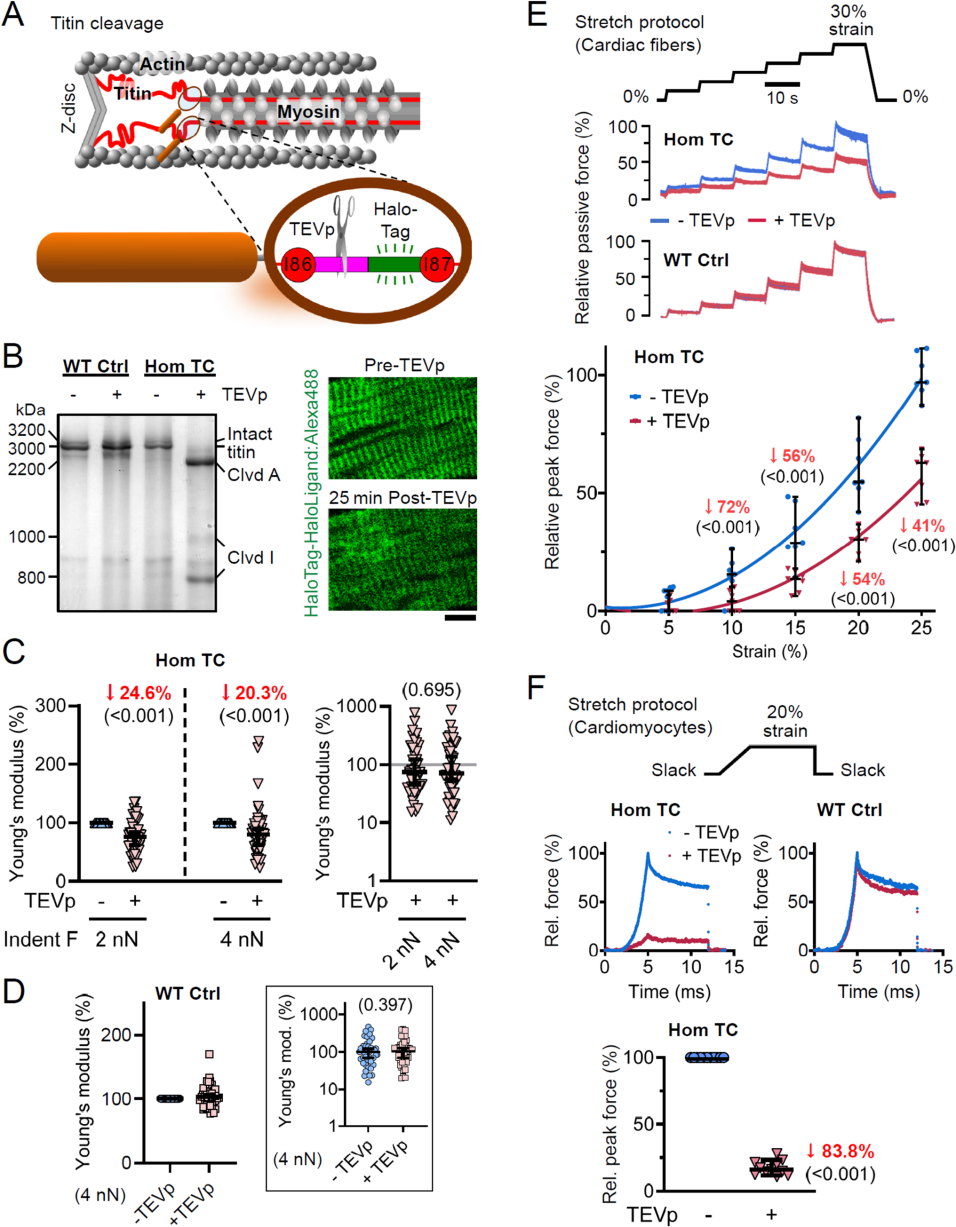
Contribution of titin springs to transverse and longitudinal passive stiffness. **A** Schematic of a Hom TC half-sarcomere illustrating the TEVp recognition site-HaloTag cassette in titin’s elastic I-band (between immunoglobulin domains I86 and I87); scissors denote TEVp cleavage. **B** Left: SDS-PAGE of permeabilized WT control (Ctrl) and Hom TC cardiac tissue after incubation in iRS + TEVp (100 mM DTT) or iRS (+ DTT) alone. Right: Hom TC cardiac section before/after TEVp, HaloTag labeled with HaloLigand-AlexaFluor-488; scale bar, 10 µm. **C** AFM nanoindentation of permeabilized Hom TC slices before/after 25 min wRS + TEVp (*n* = 94 measurements, *N* = 7 slices). Left: post/pre Young’s modulus at 2.00–2.25 nN and 4.00–4.25 nN indentation force; right: Young’s modulus (logarithmic scale) for TEVp-treated samples normalized to median pre-treatment. Data are median ± 95% CI. Analysis by LME ANOVA (treatment and force level fixed; location random). **D** AFM of WT slices (*n* = 45 measurements, *N* = 5 slices) before/after identical treatment, recorded at 4.00–4.25 nN indentation force. Inset: Young’s modulus (logarithmic scale) relative to median—TEVp condition. Data are median ± 95% CI. LME ANOVA with treatment as fixed factor, location random. **E** Passive force-extension on permeabilized Hom TC cardiac fiber bundles (*n* = 7 measurements, *N* = 5 mice) before/after 10 min iRS + TEVp, with WT fibers as controls. Top: stretch protocol; middle: representative traces (for WT, the—TEVp and + TEVp traces are superimposed); bottom: peak force of Hom TC fibers before/after treatment (median ± 95% CI) normalized to pre-treatment at 25% strain. LME ANOVA (treatment and strain fixed; fiber random); fits are second-order polynomials; significant median changes in red (*p* in parentheses). **F** Passive force of permeabilized isolated cardiomyocytes before/after 10 min iRS + TEVp. Top: stretch protocol; middle: representative traces for a Hom TC (left) and WT (right); bottom: peak force (median ± 95% CI; *n* = 11 cells, N = 5 mice) normalized to pre-treatment in Hom TC cells. One-sample t-test vs. 100; significant median change in red (*p* in parenthesis)

With titin established as the principal determinant of longitudinal passive stiffness [39, 40], we next asked whether it also contributes appreciably to transverse stiffness. To this end, we exploited the TEVp recognition site engineered into titin’s elastic region in TC mouse hearts (Fig. 3A), allowing acute, specific severing of titin springs [48]. Gel electrophoresis confirmed complete titin cleavage in Hom TC samples, whereas WT titin remained TEVp-resistant (Fig. 3B, left). Although sarcomeric cross-striation persisted under non-stress conditions, its regularity and contrast were mildly diminished post-cleavage (Fig. 3B, right). AFM nanoindentation on permeabilized TC cardiac (LV) sections before and after a 25-min TEVp treatment (sufficient to cleave 100% titins) revealed a significant reduction in transverse stiffness: Young’s modulus fell by 24.6% (17.0–38.7%) at 2.00–2.25 nN and by 20.3% (10.9–38.0%) at 4.00–4.25 nN (Fig. 3C; Fig. S2B). In contrast, TEVp had no effect on WT transverse stiffness (Fig. 3D).

To compare titin’s role in transverse versus longitudinal orientations, we stretched permeabilized LV fiber bundles in six increments from 0% (~ 1.8 µm SL) to 30% strain and recorded peak passive forces (Fig. 3E). TEVp treatment did not alter WT fibers but dramatically reduced passive forces in Hom TC bundles by 71.5% (31.5–100.0%), 56.4% (34.3–81.8%), 53.9% (27.6–67.5%), and 41.3% (21.4–55.7%) at 10%, 15%, 20%, and 25% strain, respectively (Fig. 3E, bottom) – a far greater effect than seen in the transverse direction.

Furthermore, one-step stretch assays on isolated, permeabilized LV cardiomyocytes showed that TEVp treatment reduced peak passive force at ~ 20% strain by an average of 83.8% (76.7–88.2%) in Hom TC cells, while WT cardiomyocytes remained unchanged (Fig. 3F). This pronounced decrease confirms titin’s dominant role over other cytoskeletal filament networks in determining longitudinal tensile cardiomyocyte passive stiffness [39]. Collectively, these data demonstrate that elastic titin contributes substantially more to longitudinal than to transverse passive stiffness.

### Contribution of the myosin-titin composite filament to transverse versus longitudinal passive stiffness

**Fig. 4:**
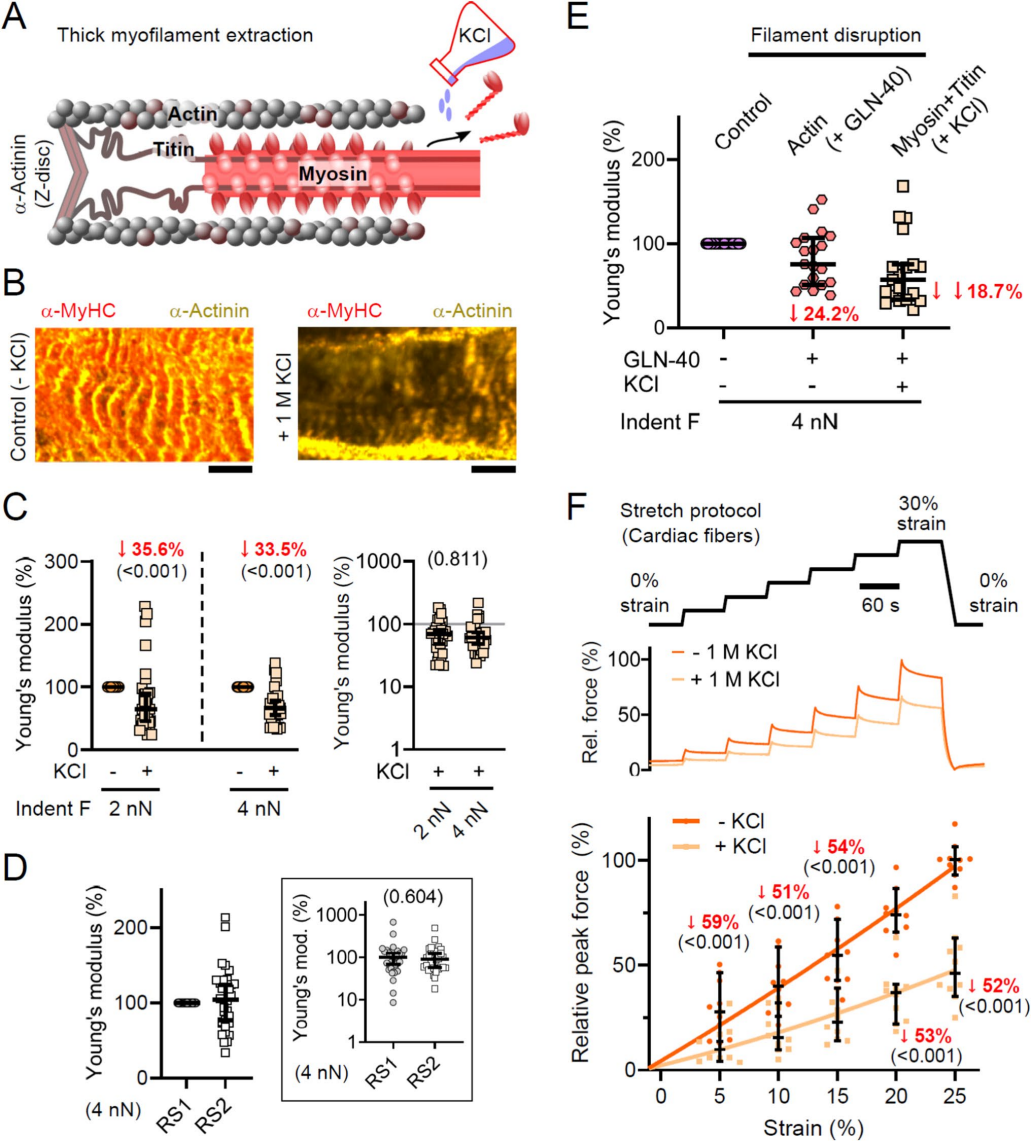
Contribution of titin-myosin composite (thick) filaments to transverse and longitudinal passive stiffness. **A** Schematic of KCl-induced thick-filament depolymerization. **B** IF images of WT cardiac tissue after 10 min in wRS alone (left) or wRS + 1 M KCl (right). Myosin (anti-α-MHC, Cy3) is shown in orange; α-actinin (EA 53, AlexaFluor-488) in yellow. Scale bars, 5 µm. **C** AFM nanoindentation of permeabilized WT slices (*n* = 63 measurements, *N* = 5 slices) before/after 10 min wRS + 1 M KCl. Left: post-/pre-treatment Young’s modulus at 2.00–2.25 nN and 4.00–4.25 nN indentation force; right: post-treatment Young’s moduli (logarithmic scale) relative to the median pre-treatment value. Data are median ± 95% CI. LME ANOVA with treatment and compression as fixed factors; location as random factor. **D** AFM of WT slices (*n* = 35 measurements, *N* = 5 slices) before/after 10 min wRS alone (RS1 vs. RS2), recorded at 4.00–4.25 nN indentation force. Inset: Young’s modulus (logarithmic scale) relative to median RS1 condition. Data are median ± 95% CI. LME ANOVA with treatment as fixed factor, location random. **E** AFM nanoindentation of WT slices (*n* = 20 measurements, *N* = 2 slices) at 4.00–4.25 nN indentation force: control (wRS), after 60 min wRS + 50 µg GLN-40, and after an additional 10 min wRS + 1 M KCl; Youngs’ moduli normalized to control condition. Data are median ± 95% CI. **F** Passive force-extension of permeabilized WT fiber bundles (n = 10 measurements, N = 6 mice) before/after 60 min wRS + 1 M KCl. Top: stretch protocol; middle: representative traces; bottom: peak force before/after treatment (median ± 95% CI) normalized to pre-treatment at 25% strain. LME ANOVA (treatment and strain fixed; fiber random); fits are second-order polynomials; significant median changes in red (*p* in parentheses)

The most abundant myofilament protein, myosin, assembles into a composite (thick) filament together with titin and myosin-binding protein C (MyBPC) [11, 61]. As a result, the passive-stiffness contributions of myosin filaments are inherently linked to those of other thick-filament components, especially titin. Many studies have taken advantage of this linkage by applying high-salt extractions (e.g., 1 M KCl) to disrupt myosin filaments, which concomitantly displaces titin’s anchorage sites. Such treatments have been used to parse titin-based versus non-titin-based contributions to passive stiffness [6, 10, 18, 23, 24, 50]. In the present work, we investigated how thick-filament disruption by 1 M KCl alters transverse and longitudinal passive stiffness (Fig. 4A) and directly compared these effects with those of targeted TEVp-mediated titin cleavage in TC cardiac fibers.

First, we assessed the impact of 1 M KCl on thick-filament integrity using IF microscopy of cardiac sections that had undergone AFM nanoindentation. Control sections displayed the expected, striated pattern and robust immunopositivity for fast myosin heavy chain (α-MyHC), the predominant myosin isoform in adult mouse cardiac sarcomeres (Fig. 4B, left). After a 10-min incubation in 1 M KCl, the myosin signal was nearly abolished, and the characteristic striations were severely disrupted, confirming effective disassembly of thick filaments (Fig. 4B, right).

Next, we conducted AFM nanoindentation on these sections before and after KCl treatment. Transverse passive stiffness decreased markedly: the median Young’s modulus was reduced by 35.6% (10.8–54.3%) at 2.00–2.25 nN and 33.5% (21.6–44.5%) at 4.00–4.25 nN (Fig. 4C; Fig. S2C), representing the largest decline observed among all treatments tested. As a control, incubation in wRS alone had no effect on transverse stiffness (Fig. 4D). Thus, high-salt extraction reduces transverse passive stiffness by approximately 1.5-fold more (Fig. 4C) than specific titin cleavage (Fig. 3C).

Because high-salt extraction could also perturb actin filaments to some degree [10, 50], we employed a sequential two-step protocol in a subset of experiments: first disrupting actin with GLN-40, then extracting thick filaments with 1 M KCl (Fig. 4E). At an indentation force of 4.00–4.25 nN, GLN-40 incubation reduced the median Young’s modulus by 24.2%, consistent with our earlier measurement. Subsequent KCl treatment produced an additional 18.7% decrease, substantially less than the > 30% reduction observed with KCl alone. Overall, the combined intervention lowered transverse passive stiffness by 42.9%, markedly below the ~ 54% predicted by the sum of individual effects. These results suggest that (i) 1 M KCl modestly disrupts actin filaments; (ii) high-salt extraction is suboptimal for isolating titin’s contribution to transverse passive stiffness; and (iii) myofilament networks interact cooperatively – analogous to a tensegrity structure – in governing transverse passive stiffness.

Finally, we assessed longitudinal passive peak forces before and after 1 M KCl treatment to compare thick-filament disruption along different axes (Fig. 4F). LV fiber bundles were stretched from 0 to 30% strain, and peak forces were recorded at 5%, 10%, 15%, 20%, and 25% strain. Following KCl treatment, forces decreased by 59.2% (37.4–76.6%), 50.5% (47.7–76.2%), 54.2% (39.1–67.5%), 52.6% (40.5–68.4%), and 51.9% (34.3–65.2%) at the respective strain levels. These values approximate those observed after specific titin cleavage, particularly at intermediate to high strains. Thus, 1 M KCl-induced thick-filament disruption provides a reasonable estimate of titin’s longitudinal stiffness contribution, especially at elevated strains.

### Overall comparison of transverse versus longitudinal passive stiffness contributions from myofilaments

**Fig. 5:**
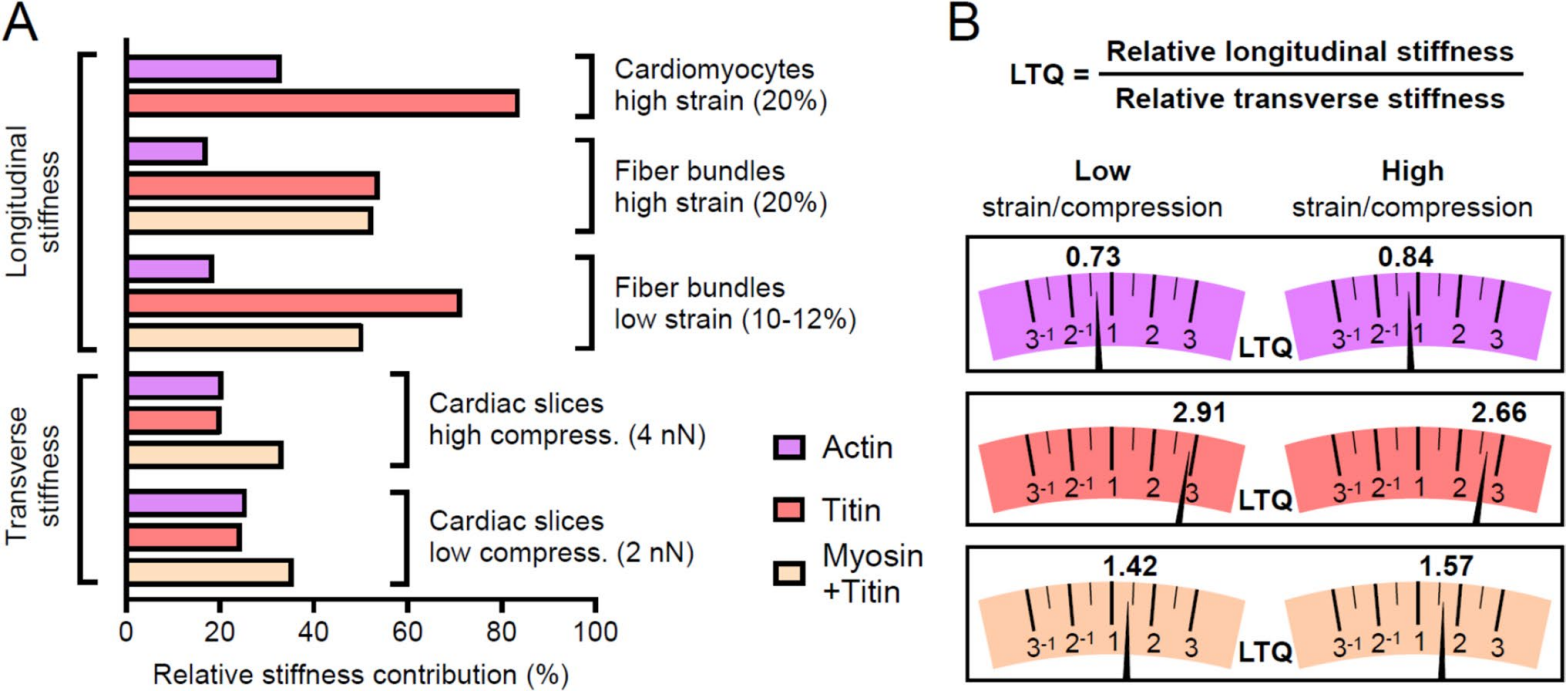
Directional contributions to passive stiffness from actin, titin, and myosin-titin composite filaments. **A** Relative stiffness of individual filament systems measured under high-strain (20%) conditions in isolated cardiomyocytes, in cardiac fiber bundles at both high (20%) and low (10–12%) strain, and by AFM nanoindentation of cardiac tissue slices subjected to high (4.00–4.25 nN) and low (2.00–2.25 nN) compressive loads. **B** Longitudinal-to-transverse stiffness quotient (LTQ) computed from the data in (A) for each myofilament type, comparing measurements in fiber bundles versus cardiac slices. Left: LTQ at low strain (10–12%) or low compression (2.00–2.25 nN) for actin, titin, and myosin-titin composite filaments; right: LTQ at high strain (20%) or high compression (4.00–4.25 nN) for the same three components

A summary of transverse versus longitudinal passive-stiffness contributions (Fig. 5) uncovers striking direction-dependent disparities across myofilament networks. Actin filaments contribute almost equally in both orientations, exhibiting only a slight transverse bias (Fig. 5A). In contrast, titin’s stiffness contribution is overwhelmingly longitudinal, exceeding twice its transverse contribution. At an indentation force of 2 nN, titin and actin each account for approximately one-quarter of total passive stiffness in the transverse axis, decreasing to about one-fifth at 4 nN. In the longitudinal orientation, the relative contribution of both actin and titin to passive stiffness appears higher in single cardiomyocytes than in fiber bundles, indirectly confirming the important role of ECM stiffness. The myosin-titin composite (thick) filaments furnish the largest transverse stiffness fraction and contribute even more along the longitudinal axis, at a level comparable to that of titin alone.

To provide a clearer perspective on these directional biases, we defined the longitudinal-to-transverse quotient (LTQ) as the ratio of longitudinal to transverse stiffness contributions measured under low and high strain/compression in both fiber bundles and cardiac slices (Fig. 5B). An LTQ of 1 denotes equal contributions. For actin, LTQs of 0.73 (low strain) and 0.84 (high strain) reveal a modest transverse predominance. In contrast, titin exhibits LTQs of 2.91 (low) and 2.66 (high), indicating a pronounced longitudinal bias. The myosin-titin composite yields intermediate values (1.42 at low and 1.57 at high strain), reflecting its relatively greater longitudinal stiffness contribution.

## Discussion

We employed AFM nanoindentation and uniaxial tensile testing to dissect transverse and longitudinal passive stiffness in permeabilized LV cardiac specimens, leveraging a genetically engineered mouse model that permits precise cleavage of titin springs. By systematically quantifying stiffness along orthogonal axes, we show that all three principal myofilament systems – actin-based thin filaments, titin springs, and the myosin-titin composite thick filaments – contribute significantly to passive myocardial mechanics, each with distinct directional biases. Longitudinal stiffness arises predominantly (> 50%) from titin, whereas transverse stiffness is more evenly distributed between actin and titin, with each contributing approximately one-quarter at low compression and one-fifth at higher compression. While thick filaments account for a substantial share of passive stiffness in both orientations, the interdependence between myosin and titin complicates their independent quantification. Notably, high-salt extraction of thick filaments approximates longitudinal titin-based stiffness with reasonable accuracy at larger strains, but markedly overestimates its transverse contribution. Collectively, our study refines the understanding of myocardial stiffness anisotropy.

To provide a concise metric for these anisotropic effects, we introduce the LTQ, the ratio of longitudinal to transverse stiffness contributions, at both low and high mechanical perturbation. Titin exhibited large LTQ values of 2.91 (low) and 2.66 (high), signifying the strong longitudinal predominance. Conversely, actin displayed modest transverse favorability (LTQ 0.73 and 0.84), and the myosin-titin composite assumed intermediate LTQs (1.42 and 1.57), demonstrating myosin’s cooperative role with titin in resisting deformations. These LTQ values provide a useful comparative framework for assessing direction-dependent mechanics across sarcomeric or other cytoskeletal and extracellular components.

Previous studies have extensively characterized longitudinal passive stiffness by dissecting, removing, or modifying specific protein structures. Strong evidence indicates that titin is the primary contributor to longitudinal stiffness in the heart [2, 18, 36, 38]. In a recent study on TC mouse hearts, we quantified titin’s role in longitudinal passive stiffness alongside other cytoskeletal elements (microtubules, actin filaments, sarcolemma-associated proteins) as well as the ECM [39]. Those findings confirmed titin’s dominant role in elastic (velocity-independent) passive force generation, whereas the viscous (velocity-dependent) component was shared among titin, microtubules, and actin, with ECM contributions becoming significant only at moderate to high stretch. Although the present work focuses on peak forces rather than separating elastic and viscous components, our observation of diminishing titin-based relative stiffness at higher strains likely reflects the increasing influence of the ECM. Similarly, the greater apparent contributions of actin and titin to passive stiffness in isolated cardiomyocytes versus cardiac fiber bundles largely reflect the absence of ECM in the former and its presence in the latter. Because high-salt extraction of myosin-titin composite thick filaments reduces longitudinal passive stiffness to an extent comparable to specific titin cleavage, our findings demonstrate that previous approaches employing high-salt extraction [6, 10, 18, 23, 24, 50] provide reasonable estimates of titin’s longitudinal contribution and can effectively separate it from ECM-based stiffness.

Transverse passive stiffness, although less extensively studied, has been measured in single myofibrils [1, 33, 42], multicellular muscle fibers, and intact cardiomyocytes [4, 43, 51, 59, 65] using AFM nanoindentation. These studies implicated actomyosin crossbridges, actin filaments, titin, microtubules, and intermediate filaments in transverse passive resistance, while thick-filament architecture, including MyBPC bridges, likely also contributes [22, 61]. However, methodological differences have complicated direct comparison of absolute stiffness values [1, 54, 66]. Our approach, which expresses stiffness values relative to baseline and samples extensively within each tissue slice, captures the intra-slice heterogeneity reported across cardiomyocyte surfaces [20, 58] and permits robust cross-condition comparisons. Crucially, our quantitative analysis demonstrates that titin is not the principal determinant of transverse stiffness; actin contributes at least as much, while thick filaments dominate, and high-salt extraction markedly underestimates the non-titin component of transverse stiffness.

Altered titin stiffness has been linked to heart failure with preserved ejection fraction (HFpEF), where increased longitudinal passive stiffness is attributed to changes in post-translational modifications of titin [36, 62, 68]. Additional cytoskeletal components, most notably microtubules [12, 55], as well as interstitial fibrosis [36], further exacerbate myocardial stiffening in HFpEF. Our findings suggest that probing transverse stiffness in HFpEF cardiac tissue may reveal novel mechanistic insights, particularly given the direction-dependent strains experienced during ventricular filling and torsion in cardiac diastole. Although HFpEF typically arises from multifactorial pathological processes, monogenic disorders such as hypertrophic cardiomyopathy (HCM) likewise augment myocardial passive stiffness [44]. HCM-associated mutations frequently target genes encoding thick- and thin-filament proteins – most prominently MyHC and MyBPC, but also actin and troponin [13, 52, 56]. While extracellular fibrosis and geometry changes contribute to passive stiffening in HCM [57, 64], sarcomeric alterations may contribute equally, potentially affecting transverse stiffness, and thus warrant further investigation.

Experimental interventions aimed at normalizing myocardial passive stiffness have demonstrated efficacy in preclinical HFpEF models, most notably via modulation of titin [5, 19, 29], yet clinical trials have frequently failed to improve patient outcomes [3, 47]. By introducing the LTQ metric and revealing consistent orientation biases among sarcomeric components, we underscore the necessity of dual-axis assessment of passive stiffness for the comprehensive preclinical evaluation of candidate therapies.

Our study is limited by the use of permeabilized tissue sections/fibers in which most membranes and associated cytoskeletal structures were removed, and by focusing exclusively on myofilament contributions to passive stiffness without direct analysis of ECM or additional intra- and extracellular elements. Moreover, juxtaposing stress–strain and stress–indentation relationships introduces interpretive ambiguity. Future work should extend LTQ analysis to intact myocardial preparations, incorporate microtubules, intermediate filaments, and ECM networks [8, 27, 28, 60], and elucidate how sarcomeric and cytoskeletal interactions govern multiscale myocardial mechanics in health and disease.

In summary, by deconvolving the anisotropic contributions of titin, thin, and thick filaments to myocardial passive stiffness, our study advances the mechanistic understanding of cardiac biomechanics and provides a quantitative framework – via the LTQ metric – for guiding targeted therapeutic strategies against stiffness-related cardiac pathologies.

## Supplementary Information

Below is the link to the electronic supplementary material.

Supplementary file1 (DOCX 176 KB)
