# Supplementary material for: Direction-dependent contributions of cardiac myofilament networks to myocardial passive stiffness reveal a major disparity for titin
Source: Basic Res Cardiol. 2025 Jun 13;120(4):761–77. doi: 10.1007/s00395-025-01119-8 (PMC12325546; doi:10.1007/s00395-025-01119-8)
Supplement: Supplementary file 1 — Supplementary file1 (DOCX 176 KB) [file 395_2025_1119_MOESM1_ESM.docx]

**Supplementary Figures**

for

**Direction-dependent contributions of cardiac myofilament networks to myocardial passive stiffness reveal a major disparity for titin**

**Authors:**

Felix A. Wagner^1^, Christine M. Loescher^1^, Andreas Unger^1^, Michel Kühn^1^, Annika J. Klotz^1^, Ivan Liashkovich^1^, Dominika Ciechanska^1^, Hermann Schillers^1^, Franziska Koser^1^, Johanna K. Freundt^1^, Anthony L. Hessel^1^, Wolfgang A. Linke^1^

**Affiliations:**

^1^Institute of Physiology II, University of Münster, Münster, Germany

**Corresponding author:**

Wolfgang A. Linke ([wlinke@uni-muenster.de](mailto:wlinke@uni-muenster.de)) ORCID: 0000-0003-0801-3773

Institute of Physiology II

University of Münster

Robert-Koch-Str. 27B

48149 Münster

**Fig. S1 Comparison of Young’s modulus in nonpermeabilized and Triton X-100 (TX-100)-permeabilized cardiac tissue slices.** Data are presented as median ± 95% CI; n = 51 measurements for nonpermeabilized (- TX-100) and n = 20 for permeabilized (+ TX-100). Measurements were made at an indentation force of 3 nN and normalized to the pre-treatment median. Statistical analysis by Mann-Whitney test

**Fig. S2 Relative contributions of actin, titin and myosin-titin composite filaments to transverse passive stiffness quantified via selective removal.** AFM nanoindentation of cardiac tissue slices, showing Young’s modulus (median ± 95% CI) at indentation force 2 nN (left) or 4 nN (right), normalized to the median pre-treatment value and overlaid with individual data points. **A**, WT slices before and after 60 min GLN-40 treatment (n = 78 measurements, N = 4 slices). **B**, Hom TC slices before and after 25 min TEVp incubation (n = 94 measurements, N = 7 slices). **C**, WT slices before and after 10 min extraction with 1 M KCl (n = 63 measurements, N = 5 slices)
